# Effect of prophylactic administration of antipyretics on the immune response to pneumococcal conjugate vaccines in children: a systematic review

**DOI:** 10.1186/s41479-021-00085-8

**Published:** 2021-04-25

**Authors:** Eleni Koufoglou, Georgia Kourlaba, Athanasios Michos

**Affiliations:** Department of Paediatric Infectious Diseases, First Department of Pediatrics, National and Kapodistrian University of Athens, “Aghia Sophia” Children’s Hospital, Thivon and Papadiamantopoulou, 11527 Athens, Greece

**Keywords:** Antipyretics, Analgesics, Pneumococcal vaccine, Conjugate, Immunogenicity, And immune response

## Abstract

**Background:**

Prophylactic administration of antipyretics at the time of immunization seems to decrease some side effects, however reduced immune responses have been reported in some studies. This systematic review aimed to investigate the effect of prophylactic use of antipyretics on the immune response following administration of pneumococcal conjugate vaccines (PCVs).

**Methods:**

A systematic review of randomized controlled trials and observational studies concerning the immune response to PCVs after antipyretic administration was performed up to November 2020 in the electronic databases of Pubmed and Scopus.

**Results:**

Of the 3956 citations retrieved, a total of 5 randomized control trials including 2775 children were included in the review. Included studies were referred to PCV10 (3 studies), PCV7 and PCV13 (one study each). The prophylactic administration of paracetamol decreased the immune response to certain pneumococcal serotypes in all included studies. The effect was more evident following primary vaccination and with immediate administration of paracetamol. Despite the reductions in antibody geometric mean concentrations, a robust memory response was observed following the booster dose. Besides, antibody titers remained above protective levels in 88–100% of participants. The use of ibuprofen, that was evaluated in two studies, did not seem to affect the immunogenicity of PCVs .

**Conclusion:**

Although the reviewed studies had significant heterogeneity in design, paracetamol administration seems to affect the immune response for certain serotypes. The clinical significance of reduced immunogenicity especially before booster dose needs further investigation.

## Introduction

*Streptococcus pneumoniae* is a major cause of morbidity and mortality worldwide, especially in children under 5 years of age, and it was estimated to cause about 294,000 deaths in children aged 1–59 months in 2015 [1, 2]. Pneumococcus is capable of colonizing the nasopharyngeal region; carriage can last from weeks to years [3]. Although colonization with pneumococcal strains is asymptomatic, it can lead to respiratory and systemic disease and is a source of spread within the community [3]. Young children are considered the most important vector for the dissemination of pneumococci within the community because of their high frequency of nasopharyngeal carriage [3].

Pneumococcal conjugated vaccines (PCVs) were licensed in 2000 and their use has a substantial impact on the burden of pneumococcal disease leading to a significant reduction in invasive pneumococcal disease through direct and indirect protection [4–6]. Different amounts of pneumococcal antibodies are required to protect against systemic disease and colonization, with higher titers needed for protection against certain serotypes and mucosal colonization [7–9].

While the contribution of pneumococcal vaccines to public health is indisputable, their administration is associated with mild adverse events such as decreased appetite, irritability, or local reactions (swelling and pain) in almost half of the recipients [10]. The incidence of some events like febrile seizures seems to occur more frequently when PCVs are co-administered with other routine vaccines [11]. There are concerns, also, that the reactogenicity of PCVs may increase by the insertion of more serotypes in the new conjugated vaccine formulations, created to deal with the “serotype replacement” phenomenon [12–14].

Although the adverse events of PCVs are mild and transient, they decrease parents’ acceptance and trust [15, 16]. Antipyretic analgesics are widely used to ameliorate vaccine adverse reactions and decrease parental anxiety, but their use has been associated with blunted vaccine immune responses to specific pneumococcal serotypes [17]. As two new PCVs (15-valent and 20-valent) are currently in phase 3 clinical trials, the effect of antipyretics on the antibody titer to specific serotypes will be crucial [18].

The objective of the present study is to systematically review the existing literature on the effect of prophylactic administration of antipyretics on the immune response of PCVs and provide a recommendation on the use of prophylactic antipyretics around the time of pneumococcal immunization.

## Materials and methods

### Literature search and study selection

In accordance with PRISMA guidelines, we conducted a systematic review to identify the impact of antipyretic use on the immune response of pneumococcal vaccination [19]. We searched the PUBMED and SCOPUS databases for English-language publications indexed through 1 November 2020. The search strategy was based on the utilization of two major groups of keywords: Paracetamol, Acetaminophen, Ibuprofen, Fever, prophylaxis, Antipyretic (Group 1) and Immune response, Antibody response, Immunity, Immunogenicity, Immunization, Immunization, Vaccination, Vaccine (Group 2). These two categories were combined by the Boolean ‘AND’ and the terms utilized within these search categories were combined by the Boolean ‘OR’. The Medical Subject Headings (MeSH) database was used for the identification of synonyms. After compilation of articles from the database and duplicate deletion, the titles and abstracts of articles were manually screened for topic relevance. A full-text review of the articles and their reference lists were then checked by two investigators. Any discordance was resolved through discussion. The reference lists of all relevant articles originally selected for inclusion in the review and relevant reviews were also searched manually to identify potentially relevant articles that were not identified by the original electronic search.

### Inclusion/exclusion criteria

Studies in English that evaluated the effect of prophylactic administration of antipyretics (paracetamol and ibuprofen) on the immunogenicity of PCVs (any type) in healthy infants/children ≥2 months by measuring serum anti-pneumococcal IgG concentrations (Geometric mean concentration-GMC) or serotype-specific opsonophagocytic activity (OPA)- Geometric mean titers (GMTs), were selected for inclusion in the present review. Randomized control studies and observational studies were eligible for inclusion as opposed to review papers, clinical guidelines, case reports, and case series. Studies concerning children with comorbidities (immunodeficiency, chronic disorders, chronic use of analgesics, or other medications) or adults were excluded. Moreover, studies where therapeutic use of antipyretics occurred were also excluded.

### Data extraction

A data extraction form was developed for this review to collect general information (authors, setting, publication year, design), participants’ baseline characteristics (number of participants, age, vaccine type and manufacturer, vaccine dosing schedule, time between receipt of vaccine and antibody testing, GMT point estimates), intervention elements (kind of antipyretic, administration schedule-time of administration) and record all significant findings.

### Methodological quality assessment

Quality assessment of studies was undertaken using the Critical Appraisal Skills Program Tool for cohort and randomized controlled trials studies (CASP) (Table 1) [20].

**Table 1:** Quality assessment of studies

Category	Item	RCT
Validity of results	**Are the results of the trial valid?**	
	1) Did the trial address a clearly focused issue?	5/5
	2) Was the assignment of patients to treatments randomized?	5/5
	3) Were all patients who entered the trial properly accounted for at its conclusion?	5/5
	4) Were patients, health workers, and study personnel ‘blind’ to treatment?	0/5
	5) Were the groups similar at the start of the trial	5/5
	6) Aside from the experimental intervention, were the groups treated equally?	5/5
Results	**What are the results?**	
	7) How large was the treatment effect?	5/5
	8) How precise was the estimate of the treatment effect?	5/5
Generalizability of results	**Will the results help locally?**	
	9) Can the results be applied to the local population, or in your context?	5/5
	10) Were all clinically important outcomes considered?	5/5
	11) Are the benefits worth harms and costs?	5/5

## Results

### Search results

**Fig. 1:**
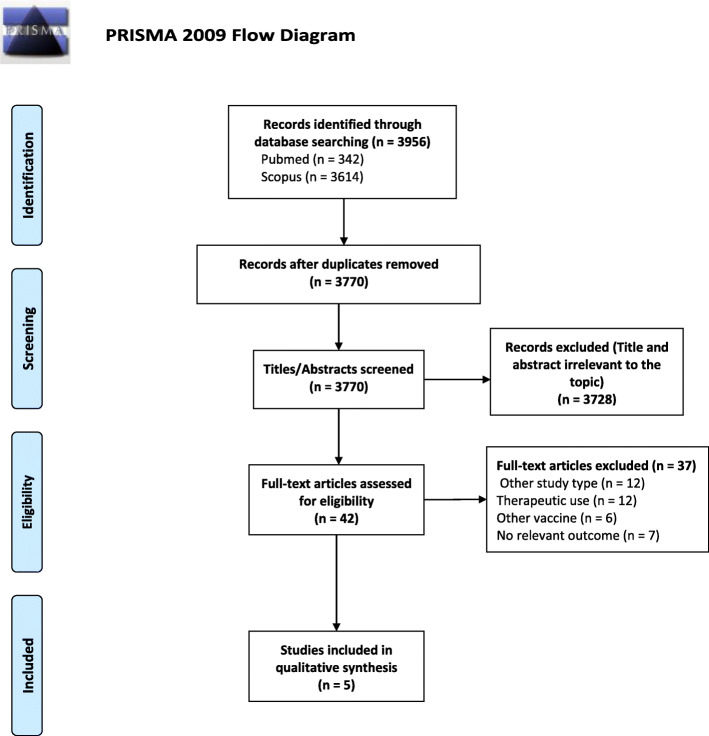
PRISMA 2009 Flow Diagram

The literature search generated a total of 3956 studies, of which 186 were duplicates. Further, 3728 studies were removed due to the irrelevance of the title and abstract to the topic of the review. A full-text review of the remaining 42 studies led to the exclusion of 37, and the identification of 5 studies which fulfilled the inclusion criteria. Details of the literature search strategy are shown in Fig. 1.

### Characteristics of the selected studies

Five studies were designed to evaluate the impact of the prophylactic use of antipyretics on the immunogenicity of pneumococcal vaccines in children [17, 21–24] (Table 2). All studies were randomized control trials (RCT), non-blinded and were performed in European Countries: three in Czech Republic, one in Poland, and one in Romania in the 2009–2017 period.

**Table 2:** Characteristics of the included studies

Author	Country, Publication year, Design	Ν	Vaccine (PCV and co-administered vaccines	Age (Primary /Booster vaccination)	Antipyretic-Intervention	Time of 1st antipyretic administration	Outcomes measured	Significant findings
Wysoski et al. 2017 [24]	Poland, 2017, RCT, non blinded	908	PCV13 (Prevenar™, Pfizer) DTaP/HBV/IPVHIb (Infarix Hexa™, GSK)	2, 3, 4 m/12 m	Paracetamol Syr (15 mg/kg/dose) Ibuprofen Syr (10 mg/kg/dose) 2 or 3doses/6–8 h for 24 h.	t:0 h or t: 6-8 h	ΙgG GMCs 1 m post-primary and booster dose (sp cut off: GMCs ≥0.35 μg/ml-non-22F ELISA) OPA GMTs in study subset	Post Primary: GMCs for all serotypes in PARA lower vs NPARA (Serotypes 3, 4, 5, 6B, 23 in IPARA, p < 0.0125). OPA GMTs detected no differences. Post booster: No effect
Falup et al. 2017 [23]	Romania, 2016, RCT, Open label, unblinded	850	PCV10 (Synflorix™, GSK) DTPa-HBV-IPV/Hib (Infanrix Hexa™, GSK)	3, 4, 5 m/12–15 m	Paracetamol Syr (15 mg/kg/dose) Ibuprofen Syr (10 mg/kg/dose) 3 doses/ 6–8 h for 24 h.	t:0 h or t: 4-6 h	ΙgG GMCs 1 m post-primary and booster dose (sp cut off: GMCs ≥0,2 μg/ml-22F-inhibition ELISA)	Post primary: % GMCs ≥0.2 μg/mL lower in IPARA and DPARA vs NPARA (highest difference for 6B serotype) GMCS for serotypes 1, 4, 5, 9 V, 14, 18C in IPARA and 1,6B in DPARA lower vs NPARA. Post booster: GMCs in IPARA-ΝPARA and the majority of participants in DPARA-IPARA lower vs NPARA-NPARA
Prymula et al. 2014 [22]	Czech Republic, 2014, RCT, Open label, unblinded	558	PCV7 (Prevenar™, Pfizer) 4CMenB (Bexsero™, Novartis) DTaP-HBV-IPV/Hib (Infanrix Hexa GSK)	2, 3, 4 m/12 m	Paracetamol Syr (10-15 mg/kg) 3 doses/ 4–6 h for 24 h.	t:0 h	ΙgG GMCs 1 m post-primary and booster dose (sp cut-off: GMCs ≥0.35 μg/ml non-22F ELISA)	GMCs for all serotypes in PARA lower vs NPARA (no statistical significance)
Prymula et al. 2013 [17]	Czech Republic, 2012, RCT, unblinded (Follow up of Prymula et al. 2009)	443	**PCV10** (Synflorix GSK)	31–44 m (2nd booster dose)	Paracetamol only at primary and 1st booster dose		ΙgG GMCs before and 7–10 days after 2nd booster dose (sp cut-off: GMCs ≥0,2 μg/ml-22F-inhibition ELISA) OPA GMTs	Before the 2nd booster: GMCs for serotypes 1, 4, 5 in PARA lower vs NPARA (borderline statistical significance). Higher OPA GMTs for serotypes 4, 5, 19F in NPARA. Post 2nd booster: GMCs with serotypes 1, 4, 9 V, 7B lower in PARA. Immunological memory robust increase in GMCs and OPA GMTs- irrespective of paracetamol use. No effect on nasopharyngeal carriage.
Prymula et al. 2009 [21]	Czech Republic, 2009, RCT, unblinded	459	**PCV10** (Synflorix GSK) DTaP/HBV/IPV/HIb (Infarix Hexa GSK) HRV (Rotarix GSK)	3, 4, 5 m./12–15 m	Paracetamol Supp (80-125 mg/dose) 3 doses/ 6–8 h for 1 24 h)	t:0 h	ΙgG GMCs 1 m post-primary and booster dose (sp cut-off: GMCs ≥0,2 μg/ml-22F-inhibition ELISA) OPA GMTs	Post primary: GMCs for all serotypes, % GMCs ≥0·20 μg/mL for 6B serotype and OPA GMTs for serotypes 1, 5, 6B lower in IPARA (*p* < 0,05) vs NPARA Post toddler dose: GMCs persisted lower in IPARA for all serotypes, apart from 19F (p < 0,05). OPA GMTs for 1, 4, 5, 6B, 19F serotypes in IPARA lower vs NPARA (p < 0,05). Similar booster response.

***Abbreviations***: ***Ν*** Number of participants, ***RCT*** Randomized control trial, ***PCV13*** Pneumococcal 13-valent Conjugated vaccine, ***PCV10*** Conjugated Pneumococcal 10-valent Conjugated vaccine, ***PCV7*** Pneumococcal 7-valent Conjugated vaccine, ***DTP-IPV-HBV-Hib*** Diphtheria (D), tetanus (T), pertussis (acellular component) (PA), hepatitis B (rDNA) (HBV), poliomyelitis (inactivated) (IPV) and *Haemophilus influenzae type b* (Hib) conjugate vaccine, ***4CMenB*** Multicomponent meningococcal capsular serogroup B protein vaccine, ***HRV*** Rotavirus vaccine, ***Syr*** Syrup, ***Supp*** Suppository, ***GMC*** Geometric mean concentration, ***GMT*** Geometric mean titer, ***ΟPA*** Opsonophagocytosis assay, ***sp cut-off*** Seroprotection cut-off, ***PARA*** Paracetamol prophylaxis group, ***IPARA*** Immediate paracetamol prophylaxis group (t:0), ***DPARA*** Delayed paracetamol prophylaxis group (t: 4-8 h), ***NPARA*** No paracetamol prophylaxis group

Included studies had a total of 2775 participants. In four studies, the study population was 2–3 months of age at the time of enrollment and 12–15 months at the time of boosting [21–24]. One clinical study [17] was a long-term follow-up study of a previous study [21], with the same children participating, who received the primary and 1st booster dose with or without paracetamol. This specific study aimed to assess the immunological memory induced after an additional dose of PCV at the age of 31–44 months (2nd booster) with no paracetamol use.

The vaccines used in the study were all conjugated pneumococcal vaccines. CRM197-PCV7 was given in one trial [22], PHiD-CV10 in three [17, 21, 23] and CRM197-PCV13 in one [24]. In all studies, the pneumococcal vaccines were co-administered with other vaccines (DTaP/HBV/IPV/Hib, Rotavirus vaccine, 4CMenB). The children received infant series immunization at 2, 3, 4 or 3, 4, 5 months of age and a booster dose at 12–15 months of age [21–24]. The second booster dose was given in children aged 31–44 months in one study [17].

All five studies examined the effect of prophylactic use of paracetamol on the immune response to PCVs [17, 21–24]. The impact of ibuprofen was evaluated in two studies [17, 22].

The participants in all five studies were randomized to receive prophylactic analgesics or receive nothing prophylactically with immunization. The antipyretic was given to children by doctors or caregivers every 4–8 h within the first 24 h following each vaccine dose of primary and booster vaccination. The administration of antipyretics started at the time of vaccination in four studies [21–24] or in two studies were administered to some participants 4–8 h after immunization [23, 24] (Immediately administration, t: 0 h or delayed administration, t: 4-8 h). Furthermore, two studies evaluated the effect of antipyretic in the case of its use only at infant doses but not in the booster [21, 23]. In one study, antipyretic was administered only for boosting [24].

The impact of prophylactic antipyretic use on the immune response to PCVs was evaluated in studies by measuring the serum anti-pneumococcal IgG concentrations and the opsonophagocytic activity of each serotype separately, before and one month after primary vaccination and before and one month after the booster vaccination. The seroprotective IgG GMC threshold was defined as GMCs ≥0.2 μg/ml (GSK’s22F inhibition ELISA) or ≥ 0.35 μg/ml (WHO’s non-22F inhibition ELISA) as well as the OPA title as ≥8, depending on the study (Table 2).

Meta-analyses of results was not feasible to perform as there was considerable clinical heterogeneity across the included trials. Firstly, different types of pneumococcal vaccines were used. The PCVs were co-administered with a different combination of routine infant vaccines. Additionally, the kind of antipyretic, the time of administration, and the number of doses also differed substantially across trials.

#### Effect of paracetamol on the immunogenicity of PCVs

For PCV10, a decreased immune response was noted in Prymula et al., and Falup et al. [21, 23]. Particularly, in Prymula et al., significantly lower antibody titers for all ten vaccine serotypes were observed one month after the primary vaccination in the group of children who received paracetamol with vaccination (t:0 h) compared with the group that did not receive paracetamol. Protective opsonophagocytic titers for pneumococcal serotypes 1, 5, and 6B were significantly lower in the prophylactic group. Although less marked, this negative effect on antibody production persisted for all vaccine serotypes except 19F, after the booster dose. Despite significantly lower antibody titers following the booster dose in the paracetamol prophylaxis group, a similar booster response (4- fold to 11-fold increase in antibody titer) was observed in both groups. The impact on the immunogenicity of vaccines after boosting was unchanged for children who did not receive paracetamol at booster vaccination, but did receive paracetamol at primary immunization [21].

A follow-up study conducted in 2013 by the same study group, with the same children participating in both studies, confirmed the transient nature of paracetamol’s impact on the immunogenicity of PCV-10 [17]. This study assessed the effect of paracetamol use during primary and 1st booster vaccination on the memory response after giving a 2nd PCV-10 booster immunization in children at the age of 4 years. They found that a strong memory response was elicited in those children, independent of the previous use of prophylactic paracetamol. The authors suggested that maybe there is no effect on memory B cells, thus the long-term immune response remains unaffected [17].

In the Falup et al. study, the immediate use of paracetamol affected antibody responses to six (1, 4, 5, 9 V, 14, and 18C) out of ten vaccine serotypes [23]. Reduced antibody titers were also found for two vaccine serotypes (1, 6B) with delayed administration. Furthermore, the percentage of children with antibody concentrations of ≥0.2 μg/ml generally tended to be lower in the immediate and delayed paracetamol groups than in the no-paracetamol group. Like the findings of the Prymula et al. study (2009), the post-booster immune response to PCV-10 appeared to be impaired when paracetamol had administered only at primary vaccination, with none given at booster vaccination. There was no effect on immunogenicity when paracetamol was given immediately only at the booster dose [23].

Regarding the immunogenicity of PCV7, in the Prymula et al. (2014) study, the pneumococcal GMC ratio suggested a negative effect of paracetamol on immunogenicity after primary as well as after the booster dose, however, immune response for all pneumococcal serotypes was considered satisfactory [22].

Regarding PCV13, in Wysocki et al. study, reduced antibody titers were found for all vaccine serotypes after primary vaccination with both immediate (t:0) and delayed (t:4-6 h) prophylactic paracetamol administration. However, this reduction was statistically significant for serotypes 3, 4, 5, 6B, and 23 (*p* < 0.0125) only when prophylactic paracetamol was given concomitantly with vaccination (t: 0 h). No impact on immune responses was observed after the booster dose [24].

In all five studies, even though there were reductions in antibody GMCs to certain pneumococcal serotypes with the use of prophylactic paracetamol, a high percentage of participants (at least 88.1% after primary and 91.7% after booster vaccination) achieved protective antibody titers level.

#### Effect of ibuprofen on the immunogenicity of PCVs

The impact of prophylactic administration of ibuprofen on the immunogenicity of PCVs was evaluated in two studies regarding PCV10 and PCV13 [23, 24]. The antibody production in both was similar regardless of the use of ibuprofen at the time of vaccination.

### Quality assessment results

All studies of the review focused on the issues of interest and used accepted methods to answer the research questions. Recruitment of participants had been conducted in an acceptable way in all RCTs studies. In all studies, patients and health workers remained unblinded to treatment. Results were well described in all studies. The acceptability and generalizability of the results were justified in all studies.

## Discussion

The present systematic review aimed to identify and report the existing literature regarding the question whether the prophylactic administration of antipyretics around the time of pneumococcal vaccination adversely affects the immunogenicity of vaccines. A great deal of interest in the topic was initially generated by Prymula et al. study (2009) that reported a significant blunting of immune responses for some vaccines when prophylactic paracetamol was given to infants during vaccination [21]. Since the last systematic review of this issue, which was published in 2014, additional studies were published regarding PCV10 and PCV13 [23–25].

In the present analysis, a negative effect of prophylactic paracetamol on antibody response for some vaccine pneumococcal serotypes was observed in all included RCTs. This negative effect was more evident if paracetamol was used with primary immunization. Despite the initial reduction in pneumococcal antibodies concentration, a robust memory response was elicited after booster vaccination. In addition, no effect of prophylactic paracetamol administration was noticed when paracetamol was given only at the booster dose. Finally, it is remarkable that most participants developed protective antibody titers (≥0,2 μg/ml, ή ≥0,35 μg/ml), which calls the clinical significance of the findings into question.

The effect of paracetamol on the immunogenicity of PCVs, in the studies included in this review, was mainly detected when the first dose was given concomitantly with vaccination. In contrast, after delayed paracetamol administration (usually 4–6 h following vaccination), which was evaluated only in two studies, little or no impact was observed.

This time-related effect on antibody induction was also observed for other vaccine antigens, like in the Doedee et al. study, where a decrease in the immune response against Hepatitis B antigen (HBV) was significant only with immediate administration of paracetamol but not with delayed [26]. Results from studies regarding multicomponent vaccines indicate that when antipyretics were given as a treatment (usually ≥6 h), there was no effect on the immunogenicity of antigens, which can support the hypothesis that delayed administration of antipyretics may not interfere with the immune response to vaccines [27–29].

The above observations highlight the notion that the relationship between antigen exposure and the timing of antipyretic administration seems to play a vital role in modifying the immune system response to vaccination. Interference of analgesics mainly with innate (primary) immune responses, is a possible explanation of this time-dependent effect of antipyretics on the immunogenicity of vaccines. The early stages of the immune system’s stimulation (e.g., the migration of monocytes at the site of injection and the presentation of the antigen to CD4+ helper cells by dendritic cells), begin immediately after vaccination and progress to the first 24 h thereafter, a period that coincides with the antipyretic administration in the studies [30]. In addition, the fact that memory responses remain strong independently of antipyretic use suggests that the memory cell reservoir - which takes several weeks or months to form and mature - and its mobilization is unaffected by analgesic use [30]. As the mechanism underlying the decrease in immune response is unexplored, this set of observations can direct the focus of future research.

Even though the exact mechanism by which antipyretic interfere with the immune system is not known, several studies have already shown their ability to do so at various points. Paracetamol can affect the immune response by lowering the level of glutathione in the liver, which is associated with lymphocyte activation, as well as with its action as a selective COX2 inhibitor [31]. The action of paracetamol on the immune system is also proven by observational studies in which its use led to prolonged rhinovirus, chickenpox, and malaria infections, but also adversely affected mortality in patients with sepsis [32–35].

Ibuprofen did not affect the immunogenicity of pneumococcal vaccines in our review, although only two studies reported on this issue [23, 24]. In contrast, a negative impact on the immunogenicity of vaccines with the use of ibuprofen was reported by Wysoski et al., and Falup et al., for the Pertussis FHA, Tetanus, and HBSAbs antigens [23, 24].

According to in vitro studies, the use of ibuprofen and aspirin may affect leukocyte migration at the site of inflammation and reduce neutrophil adhesion by reducing the expression of VCAM-1 and ICAM-1 [36]. They also affect the antigen-presenting capacity of dendritic cells, which plays a key role in the development of primary immune responses [37]. NSAIDs, as inhibitors of cyclooxygenase enzymes, affect the production of antibodies when they act on B cell cultures [38, 39].

Antipyretics to ameliorate the adverse events of vaccination were widely used by parents and doctors for several decades, with no studies reporting a negative effect on the vaccine’s immunogenicity until 2009 [40, 41]. Furthermore, the National Health System of England has recently published a protocol to guide paracetamol administration around MenB vaccination [42].

The fact that despite the widespread practice of antipyretic use around immunization time, vaccines are highly effective in the population may question the clinical significance of these findings. The reduction in antibody production, however, suggests that an optimal immune response is obtained without prophylactic antipyretics. The use of antipyretics may modify the protective role of PCVs in certain situations where higher titers of antibodies are required, such as for protection against otitis media infections or the reduction of nasopharyngeal carriage [7, 8]. It also remains unknown how important this effect could be when added in situations affecting the immune response to vaccines such as immunosuppression, chronic diseases, extreme age, daily habits (smoking, alcohol, and stress), genetic factors or co-administration of vaccines [43].

The findings of this systematic review provide evidence in support of the recently updated guidelines by the American Academy of Pediatrics (AAP), World Health Organization (WHO), American Committee on Immunization Practices (ACIP) and other national guidelines for the use of antipyretics at the time of vaccination. These guidelines recommend against the use of antipyretics prophylactically around the time of immunization, even for children with a positive history of seizures, due to the risk of reduced immunogenicity of vaccines. On the contrary, therapeutic administration is permitted [44–47].

### Limitations

The results of the review should be interpreted with caution. Heterogeneity was recorded in the studies regarding the type of vaccine and antipyretic used as well as the timing of antipyretic administration. The fact that all studies were conducted in developed countries and concern healthy children makes it difficult to generalize the results to developing countries as well as to other groups of the population such as e.g., immunocompromised, or chronic users of anti-inflammatory drugs. Furthermore, we should acknowledge the possibility of publication bias since only studies written in the English language are included in the review. The small number of studies and the heterogeneity in their design prevented us from carrying out a meta-analysis.

## Conclusions

Although the use of antipyretics, especially paracetamol, at the time of vaccination appears to reduce the side effects of vaccines, there is a decrease in antibody titers for some PCV antigens. This may raise doubts about the practice of antipyretic administration around vaccination time. This effect differs depending on the antipyretic agent used and may have a time-dependent administration component. The clinical significance of these findings is questionable, especially between primary and booster doses where antibody titers wane. However, after the booster doses, most participants developed protective antibody titers against vaccine antigens.

The small number of studies included in the above review does not allow us to draw certain conclusions. Especially after the near future possible introduction of 15 and 20-valent PCVs, questions regarding the effect of antipyretics on the immunogenicity of vaccines concerning the dose, frequency, and timing should be clarified. More well-designed future studies need to be conducted to provide clear evidence regarding the underlying mechanism and the possible association of immunogenicity with the type of antipyretics and the time of administration.

## Data Availability

The data that support the findings of this study are available from the corresponding author upon request.

## Abbreviations

PCV: Pneumococcal conjugate vaccination
MeSH: Medical Subject Headings
GMC: Geometric mean concentration
OPA: Serotype-specific opsonophagocytic activity
AAP: American Academy of Pediatrics
WHO: World Health Organization
ACIP: American Committee on Immunization Practices
RCT: Randomized control trials
